# Supplementary material for: Modelling Skylarks (Alauda arvensis) to Predict Impacts of Changes in Land Management and Policy: Development and Testing of an Agent-Based Model
Source: PLoS One. 2013 Jun 6;8(6):e65803. doi: 10.1371/journal.pone.0065803 (PMC3675089; doi:10.1371/journal.pone.0065803)
Supplement: Supporting Information S4 — The skylark ODdox as a zipped archive. (ZIP) [file pone.0065803.s004.zip › Skylark_ODdox/functions_func_0x73.html]

ALMaSS Skylark ODdox: Class Members - Functions


ALMaSS Skylark ODdox
 2.0


- Main Page
- Related Pages
- Classes
- Files

- Class List
- Class Index
- Class Hierarchy
- Class Members

- All
- Functions
- Variables
- Typedefs

- a
- b
- c
- d
- e
- f
- g
- h
- i
- j
- k
- l
- m
- n
- o
- p
- r
- s
- t
- u
- v
- w
- y
- z
- ~

### - s -

- Saltwater()
  : Saltwater
- SandDune()
  : SandDune
- SavePPM()
  : Pesticide
- ScalablePositionMap()
  : ScalablePositionMap
- Scrub()
  : Scrub
- SensibleCopy()
  : Skylark\_Female
  , Skylark\_Male
- set()
  : CfgInt
  , CfgFloat
  , CfgBool
  , CfgStr
- SetArea()
  : LE
- SetBLtype()
  : BaitLocation
- SetBorder()
  : LE
- SetCanGrow()
  : EdgeGrowingPoints
- SetCentroid()
  : LE
- SetCfgBool()
  : Configurator
- SetCfgFloat()
  : Configurator
- SetCfgGatekeeper()
  : Configurator
- SetCfgInt()
  : Configurator
- SetCfgStr()
  : Configurator
- SetCopyTreatment()
  : LE
- SetCountryDesignation()
  : LE
- SetCropData()
  : LE
  , VegElement
- SetCropDataAll()
  : LE
  , VegElement
- SetElementType()
  : LE
- SetF\_Mig\_Mort()
  : Skylark\_Population\_Manager
- SetFarmNumber()
  : Farm
- SetFile()
  : probe\_data
- SetFileType()
  : LE
- SetFirstYear()
  : Calendar
- SetGrowthPhase()
  : LE
  , VegElement
- SetHerbicideDelay()
  : LE
- SetHigh()
  : LE
- SetInsectPop()
  : LE
  , VegElement
- SetLastTreatment()
  : LE
- SetLastYear()
  : Calendar
- SetLESignal()
  : Landscape
- SetM\_Mig\_Mort()
  : Skylark\_Population\_Manager
- SetMap()
  : RodenticideManager
- SetMapIndex()
  : LE
- SetMapTwin()
  : RodenticideManager
- SetMapTwinMult()
  : RodenticideManager
- SetMapTwinP()
  : RodenticideManager
- SetMapValid()
  : LE
- SetMapValue()
  : MovementMap
  , MovementMap16
  , IDMap
  , SimplePositionMap
  , ScalablePositionMap
  , PositionMap
- SetMapValue0()
  : MovementMap
- SetMapValue1()
  : MovementMap
- SetMapValue2()
  : MovementMap
- SetMapValue3()
  : MovementMap
- SetMass()
  : BaitLocation
- SetMaxX()
  : LE
- SetMaxY()
  : LE
- SetMConstants()
  : LE
- SetMDates()
  : LE
- SetMgtLoopDetectCount()
  : LE
- SetMgtLoopDetectDate()
  : LE
- SetMinX()
  : LE
- SetMinY()
  : LE
- SetMownDecay()
  : LE
- SetNestPossibility()
  : skTTerritory
- SetNoProbes()
  : Population\_Manager
- SetOldDays()
  : LE
- SetOwner()
  : LE
- SetPesticideCell()
  : LE
- SetPoison()
  : LE
- SetPoly()
  : LE
- SetPolyMaxMinExtents()
  : Landscape
- SetQuality()
  : skTTerritory
- SetRotIndex()
  : LE
- SetSignal()
  : LE
- SetSoilType()
  : LE
- SetstartDay()
  : BaitLocation
- SetSubType()
  : LE
- SetTramlinesDecay()
  : LE
- SetUnsprayedMarginPolyRef()
  : LE
- SetUseFlag()
  : BaitLocation
- SetValidXY()
  : LE
- SetValue()
  : BinaryMapBase
- SetVegBiomass()
  : LE
- SetVegHeight()
  : LE
  , VegElement
- SetVegNum()
  : CropData
- SetVegPatchy()
  : LE
  , VegElement
- SetVegStore()
  : LE
- SetVegType()
  : LE
  , VegElement
- SetVirtualDiameter()
  : skTTerritory
- SetWarnLevel()
  : MapErrorMsg
- SetX()
  : BaitLocation
  , TAnimal
- SetY()
  : BaitLocation
  , TAnimal
- ShouldPlaceBait()
  : RodenticideManager
- ShowIdType()
  : Configurator
- ShrinkList()
  : EdgeGrowingPoints
- Shuffle()
  : Population\_Manager
- Shuffle\_or\_Sort()
  : Population\_Manager
- SimpleEvent()
  : Crop
- SimplePositionMap()
  : SimplePositionMap
- SkTerritories()
  : SkTerritories
- skTTerritory()
  : skTTerritory
- Skylark\_Adult()
  : Skylark\_Adult
- Skylark\_Base()
  : Skylark\_Base
- Skylark\_Clutch()
  : Skylark\_Clutch
- Skylark\_Female()
  : Skylark\_Female
- Skylark\_Male()
  : Skylark\_Male
- Skylark\_Nestling()
  : Skylark\_Nestling
- Skylark\_Population\_Manager()
  : Skylark\_Population\_Manager
- Skylark\_PreFledgeling()
  : Skylark\_PreFledgeling
- SkylarkEvaluation()
  : Landscape
- SleepAllDay()
  : Farm
- SmallRoad()
  : SmallRoad
- SortState()
  : Population\_Manager
- SortStateR()
  : Population\_Manager
- SortX()
  : Population\_Manager
- SortXIndex()
  : Population\_Manager
- SortY()
  : Population\_Manager
- SpeciesSpecificReporting()
  : Population\_Manager
- Spiral()
  : Skylark\_Female
- Spiral2()
  : Skylark\_Female
- Split()
  : SkTerritories
- SpringHarrow()
  : Farm
- SpringPlough()
  : Farm
- SpringRoll()
  : Farm
- SpringSow()
  : Farm
- st\_Arriving()
  : Skylark\_Female
  , Skylark\_Male
- st\_AttractingAMate()
  : Skylark\_Male
- st\_BuildingUpResources()
  : Skylark\_Female
- st\_CaringForYoung()
  : Skylark\_Female
  , Skylark\_Male
- st\_Developing()
  : Skylark\_Clutch
  , Skylark\_Nestling
  , Skylark\_PreFledgeling
- st\_Dying()
  : Skylark\_Clutch
  , Skylark\_Nestling
  , Skylark\_PreFledgeling
  , Skylark\_Female
  , Skylark\_Male
- st\_EggHatching()
  : Skylark\_Female
- st\_Emigrating()
  : Skylark\_Female
  , Skylark\_Male
- st\_Finding\_Territory()
  : Skylark\_Female
- st\_FindingTerritory()
  : Skylark\_Male
- st\_Floating()
  : Skylark\_Female
  , Skylark\_Male
- st\_Flocking()
  : Skylark\_Female
  , Skylark\_Male
- st\_FollowingMate()
  : Skylark\_Male
- st\_GivingUpTerritory()
  : Skylark\_Female
- st\_Hatching()
  : Skylark\_Clutch
- st\_Immigrating()
  : Skylark\_Female
  , Skylark\_Male
- st\_Incubating()
  : Skylark\_Female
- st\_Laying()
  : Skylark\_Female
- st\_MakingNest()
  : Skylark\_Female
- st\_Maturing()
  : Skylark\_Nestling
  , Skylark\_PreFledgeling
- st\_PreparingForBreeding()
  : Skylark\_Female
- st\_ScaringOffChicks()
  : Skylark\_Male
- st\_StartingNewBrood()
  : Skylark\_Female
- st\_StoppingBreeding()
  : Skylark\_Female
- st\_TempLeavingArea()
  : Skylark\_Female
  , Skylark\_Male
- StartDeveloping()
  : Skylark\_Clutch
- StartValid()
  : CropData
- Step()
  : TALMaSSObject
  , TAnimal
  , Skylark\_Clutch
  , Skylark\_Nestling
  , Skylark\_PreFledgeling
  , Skylark\_Female
  , Skylark\_Male
- StepFinished()
  : Population\_Manager
- StepOneValid()
  : Landscape
- StoneWall()
  : StoneWall
- StoreLAItotal()
  : VegElement
  , LE
- StrawChopping()
  : Farm
- Strigling()
  : Farm
- StriglingSow()
  : Farm
- StubbleHarrowing()
  : Farm
- Suburban()
  : Suburban
- Supply\_BreedingAttempts()
  : Skylark\_Female
- Supply\_BreedingSuccess()
  : Skylark\_Female
- Supply\_F\_Owner()
  : SkTerritories
- Supply\_m\_Location\_x()
  : TAnimal
- Supply\_m\_Location\_y()
  : TAnimal
- Supply\_NestTime()
  : Skylark\_Female
- Supply\_Owner()
  : SkTerritories
- Supply\_quality()
  : SkTerritories
- Supply\_size()
  : SkTerritories
- Supply\_terr()
  : SkTerritories
- Supply\_Territory()
  : Skylark\_Male
- Supply\_TerritoryQual()
  : Skylark\_Male
- Supply\_x()
  : SkTerritories
- Supply\_y()
  : SkTerritories
- SupplyBroodAge()
  : Skylark\_Male
- SupplyBroodSize()
  : Skylark\_Male
- SupplyBroodWeight()
  : Skylark\_Male
- SupplyCountryDesig()
  : Landscape
- SupplyCovPosx()
  : Population\_Manager
- SupplyCovPosy()
  : Population\_Manager
- SupplyDayDegrees()
  : Landscape
- SupplyDayInMonth()
  : Landscape
- SupplyDayInYear()
  : Landscape
- SupplyDaylength()
  : Landscape
- SupplyDeadBiomass()
  : Landscape
- SupplyElementSubType()
  : Landscape
- SupplyElementType()
  : Landscape
- SupplyElementTypeCC()
  : Landscape
- SupplyElementTypeFromVector()
  : Landscape
- SupplyEMi()
  : Skylark\_Population\_Manager
- SupplyF\_Mig\_Mort()
  : Skylark\_Population\_Manager
- SupplyFarmArea()
  : Landscape
- SupplyFarmIntensity()
  : Landscape
- SupplyFarmOwner()
  : Landscape
- SupplyFarmOwnerIndex()
  : Landscape
- SupplyFarmOwnerRef()
  : TAnimal
- SupplyFarmType()
  : Landscape
- SupplyGlobalDate()
  : Landscape
- SupplyGlobalRadiation()
  : Landscape
- SupplyGrazingPressure()
  : Landscape
- SupplyGrazingPressureVector()
  : Landscape
- SupplyGreenBiomass()
  : Landscape
- SupplyHasTramlines()
  : Landscape
- SupplyInsects()
  : Landscape
- SupplyIsGrass()
  : Landscape
- SupplyIsNestValid()
  : SkTerritories
- SupplyJustMown()
  : Landscape
- SupplyJustMownVector()
  : Landscape
- SupplyJustSprayed()
  : Landscape
- SupplyJustSprayedVector()
  : Landscape
- SupplyLAGreen()
  : Landscape
- SupplyLargestPolyNumUsed()
  : Landscape
- SupplyLastTreatment()
  : Landscape
- SupplyLATotal()
  : Landscape
- SupplyLECount()
  : Landscape
- SupplyLENext()
  : Landscape
- SupplyLEPointer()
  : Landscape
- SupplyLEReset()
  : Landscape
- SupplyLESignal()
  : Landscape
- SupplyListIndexSize()
  : Population\_Manager
- SupplyListName()
  : Population\_Manager
- SupplyListNameLength()
  : Population\_Manager
- SupplyListSize()
  : Population\_Manager
- SupplyLocXY()
  : Population\_Manager
- SupplyM\_Mig\_Mort()
  : Skylark\_Population\_Manager
- SupplyMagicMapP()
  : Landscape
- SupplyMeanTemp()
  : Landscape
- SupplyMonth()
  : Landscape
- SupplyMyClutch()
  : Skylark\_Female
- SupplyNestLoc()
  : Skylark\_Male
- SupplyNestPosition()
  : SkTerritories
- SupplyNestValid()
  : Skylark\_Male
- SupplyNoFemaleOccupied()
  : SkTerritories
- SupplyNoHabitatRefs()
  : Skylark\_Male
- SupplyNoMaleOccupied()
  : SkTerritories
- SupplyNoTerritories()
  : SkTerritories
  , Skylark\_Population\_Manager
- SupplyNumberOfPolygons()
  : Landscape
- SupplyPegPosx()
  : Population\_Manager
- SupplyPegPosy()
  : Population\_Manager
- SupplyPesticide()
  : Pesticide
  , Landscape
  , Pesticide
  , Landscape
- SupplyPesticideCell()
  : Landscape
- SupplyPesticideType()
  : Landscape
- SupplyPolygonAreaVector()
  : Landscape
- SupplyPolygonRef()
  : TAnimal
- SupplyPolyRef()
  : Landscape
- SupplyPolyRefCC()
  : Landscape
- SupplyPolyRefIndex()
  : Landscape
- SupplyPolyRefVector()
  : Landscape
- SupplyPosition()
  : TAnimal
- SupplyRain()
  : Landscape
- SupplyRainPeriod()
  : Landscape
- SupplyRoadWidth()
  : Landscape
- SupplyRodenticide()
  : Landscape
- SupplyShouldSpray()
  : Landscape
- SupplySimAreaHeight()
  : Landscape
- SupplySimAreaWidth()
  : Landscape
- SupplySimH()
  : Population\_Manager
- SupplySimW()
  : Population\_Manager
- SupplySkScrapes()
  : Landscape
- SupplySnowcover()
  : Landscape
- SupplyState()
  : Population\_Manager
- SupplyStateList()
  : Population\_Manager
- SupplyStateNames()
  : Population\_Manager
- SupplyStateNamesLength()
  : Population\_Manager
- SupplyStepSize()
  : Population\_Manager
- SupplyTemp()
  : Landscape
- SupplyTempPeriod()
  : Landscape
- SupplyTrafficLoad()
  : Landscape
- SupplyTreeAge()
  : Landscape
- SupplyTreeHeight()
  : Landscape
- SupplyUnderGrowthWidth()
  : Landscape
- SupplyValidX()
  : Landscape
- SupplyValidY()
  : Landscape
- SupplyVegAge()
  : Landscape
- SupplyVegBiomass()
  : Landscape
- SupplyVegBiomassVector()
  : Landscape
- SupplyVegCover()
  : Landscape
- SupplyVegCoverVector()
  : Landscape
- SupplyVegDensity()
  : Landscape
- SupplyVegDigestability()
  : Landscape
- SupplyVegDigestabilityVector()
  : Landscape
- SupplyVegHeight()
  : Landscape
- SupplyVegHeightVector()
  : Landscape
- SupplyVegPatchy()
  : Landscape
- SupplyVegType()
  : Landscape
- SupplyVegTypeVector()
  : Landscape
- SupplyVersion()
  : Landscape
- SupplyWeedBiomass()
  : Landscape
- SupplyWind()
  : Landscape
- SupplyWindDirection()
  : Landscape
- SupplyWindPeriod()
  : Landscape
- SupplyYear()
  : Landscape
- SupplyYearNumber()
  : Landscape
- Swathing()
  : Farm
- SynInsecticideTreat()
  : Farm


- Generated on Thu Jan 10 2013 13:15:39 for ALMaSS Skylark ODdox by
   1.8.1.1
